# Soil Dehydrogenases as an Indicator of Contamination of the Environment with Petroleum Products

**DOI:** 10.1007/s11270-015-2642-9

**Published:** 2015-10-11

**Authors:** Grażyna Kaczyńska, Agata Borowik, Jadwiga Wyszkowska

**Affiliations:** Department of Microbiology, University of Warmia and Mazury in Olsztyn, Plac Lodzki 3, 10-727 Olsztyn, Poland

**Keywords:** Soil dehydrogenases, Soil biostimulation, Petroleum products, Remediation

## Abstract

The aim of the research was to compare the effects of various petroleum products, biodiesel, diesel oil, fuel oil and unleaded petrol on soil dehydrogenases, and to evaluate biostimulation with compost and urea in the restoration of homeostasis of the soil contaminated with these products. The obtained results allowed for defining the weight of dehydrogenases in monitoring of the environment subjected to pressure from petroleum hydrocarbons. The studies were carried out under laboratory conditions for 180 days, and loamy sand was the soil formation used in the experiment. The petroleum products were used in the following amounts: 0, 2, 4, 8 and 16 g kg^−1^ DM of soil. Indices of the influence of the petroleum product and the stimulating substance on the activity of dehydrogenases were calculated. It was proved that the petroleum products affect soil dehydrogenases in various ways. Biodiesel, diesel oil and fuel oil stimulate these enzymes, while petrol acts as an inhibitor. Among the substances tested regarding biostimulation of soils contaminated with petroleum products, compost is definitely more useful than urea, and therefore, the former should be used for the remediation of such soils. Stimulation of dehydrogenases by compost, both in contaminated and non-contaminated soils, proves that it may accelerate microbiological degradation of petroleum-derived contaminants.

## Introduction

Preservation of the biological equilibrium in the soil depends on numerous factors, which may be divided into chemical, physical and biological factors. The last group of parameters is particularly susceptible to modifications caused by any disturbance occurring in the soil and water environment. Enzymatic activity of the soil and proliferation of soil microorganisms are the best indicators of the stability and fertility of soil ecosystems. This is because of the immediate response of the biochemical activity of the soil to any disturbance of the environment. The soil environment is a source of an immense pool of enzymes. It includes representatives of every enzyme class, i.e. oxidoreductases, hydrolases, isomerases, ligases, liases and transferases. They all perform key functions in the process of the conversion of organic substances and energy (Gu et al. 2009). Soil dehydrogenases (EC 1.1.1.) are the main representatives of the oxidoreductase class.

Among all enzyme groups of the soil environment, dehydrogenases belong to one of the most important ones, because they occur in all living microorganism cells (Moeskops et al. 2010). As they are closely connected with microbiological redox processes (Gu et al. 2009), dehydrogenases are often considered as an index of the general microbiological activity of the soil (Moeskops et al. 2010). The significance of dehydrogenases as a pollution indicator is additionally supported by their lack of ability to accumulate in the extracellular environment. The role of dehydrogenases consists in the biological oxidation of organic matter in the soil by hydrogen transfer from the organic substrate to inorganic acceptors (Zhang et al. 2010). It should be emphasised that in the environment, many specific types of dehydrogenases occur, differing in terms of, among others, the coenzyme type (nicotinamide adenine dinucleotide, nicotinamide adenine dinucleotide phosphate or flavin adenine dinucleotide). Thanks to the ability to carry out dehydrogenation or hydrogenation, dehydrogenases are involved in the enzymatic systems of all living microorganisms (Subhani et al. 2001). Therefore, soil dehydrogenases are reported as an indicator of microbiological redox systems. Brzezińska et al. (2001) ascertained that these enzymes may use not only oxygen molecules as electron acceptors but also other compounds which occur in cells of anaerobic microorganisms. Thus, the activity of dehydrogenases reflects the rate of transformations occurring in the soil.

Petroleum products cause great devastation in soil and water ecosystems (Yeung et al. 2011). This results from the fact that these substances are mixtures of organic compounds with a low bioavailability, often described as potentially carcinogenic and mutagenic (Janbandhu and Fulekar 2011; Souza et al. 2014). Yeung et al. (2011) report that 1 dm^3^ of oil pollutes 1 million dm^3^ of water because of its ability to form a thin film with a surface area of 1000 m^2^. This film prevents gas exchange and blocks the access of sunlight. Thus, it is not surprising that petroleum hydrocarbons belong to compounds considered highly toxic for soil ecosystems and human health (Albert and Tanee 2011).

In respect of chemical character, hydrocarbons may be included among bioresistant organic compounds (Bio-ROCs), which are able to completely or partially inhibit the metabolism of aerobic microorganisms. This is a consequence of the poor solubility of these substances in water, limited adsorbability and complex molecular structure (Semrany et al. 2012). As a consequence of their specific chemical features, such compounds are reduced by few enzymes (Lloret et al. 2010). As a result of the high potential of petroleum hydrocarbons to accumulate in the soil environment, together with their resistance to biodegradation, these compounds cause substantial changes in biological parameters, such as enzymatic activity and abundance of microbes (Pérez-Leblic et al. 2010, Zhan et al. 2010). One technique attempting to accelerate the natural degradation of petroleum products by the introduction of nutrients is called biostimulation (Chemlal et al. 2012).

The aim of such actions consists in the stimulation of native microorganisms, which become resistant to the already occurring disturbances. Currently, it is a fast-growing research theme. This dependence is probably affected not only by the type of the soil (Aspray et al. 2008) or the kind of contaminant (Dilly et al. 2011) but also by the enzymatic system of the microbes. Lamy et al. (2013) suggest that the biostimulation is favourable only when, for the mineralisation of the compound used for it, the cell may utilise the enzymatic complexes of metabolic pathways used for degradation of the contaminating compounds.

The aim of the research was to compare the effects of various petroleum products, biodiesel, diesel oil, fuel oil and unleaded petrol on soil dehydrogenases, and to evaluate biostimulation with compost and urea in the restoration of the homeostasis of soil contaminated with these products. The obtained results allowed for defining the weight of dehydrogenases in the monitoring of environments subjected to pressure from petroleum hydrocarbons.

## Material and Methods

### Soil

In the research, soil from the Tomaszkowo Didactic and Experimental Station (NE part of Poland, 53.7161° N, 20.4167° E) was used. The soil material was collected from the topsoil of typical brown earths (Eutric Cambisol). According to the graining classification of the US Department of Agriculture, it was soil with a granulometric composition of loamy sand, with characteristics shown in Table 1.

**Table 1:** Physicochemical properties of the soil

Properties	Unit	Value
The percentage of the fraction (*d*)	2.00 ≥ *d* > 0.05 mm	79.43
	0.05 ≥ *d* > 0.002 mm	18.82
	*d* ≤ 0.002 mm	1.75
pH_KCl_		4.69
HAC	mM(+) kg^−1^ DM of soil	24.10
EBC		32.70
CEC		56.80
BS	%	57.57
C_org_	g kg^−1^ DM of soil	11.60
N_c_		1.39
K_a_	mg kg^−1^ DM of soil	129.48
P_a_		98.56
Mg_a_		45.00

*HAC* hydrolytic acidity, *EBC* sum of exchangeable cations, *CEC* cation exchange capacity, *BS* base saturation, *org* organic, *c* total, *a* available

### Petroleum Products

In the experiment, the following petroleum products were used: biodiesel, diesel oil, fuel oil and unleaded petrol. Their basic properties are shown in Table 2.

**Table 2:** Properties of petroleum products

Parameter	Unit	Biodiesel (Bd)	Diesel (D)	Fuel oil (Fo)	Unleaded petrol (P)
		Value			
Number of carbon atoms	–	C_6_–C_12_	C_9_–C_25_	C_9_–C_20_	C_5_–C_12_
Density	g cm^−3^	0.88–0.89	0.82–0.85	0.80–0.91	0.72–0.78
Calorific value	MJ kg^−1^	37–39	42–45	42–45	42–44
Cetane number	–	51	55	55	b.d.
PAH content	%	dl	11	dl	20
Content of solid impurities	mg kg^−1^	24	24	24	dl
Sulphur content	mg kg^−1^	10	10	2–3	10
Ignition point	°C	170	56	56–62	−51
Boiling point	°C	>350	175–180	180–360	30–210
Self-ignition point	°C	260	260	270	350
Acute toxicity by inhalation (DNEL)	mg m^−3^	dl	4300	4300	2800
	15 min^−1^				

*dl* data lack

### Substances Used for Biostimulation

Compost and urea were used for the biostimulation of the soil contaminated with petroleum products. The compost was manufactured by a company NOLET (Poland) as a result of the aerobic composting of conifer sawdust and turkey litter. The organic carbon content in the compost amounted 402.0 g kg^−1^, to total nitrogen 23.1 g kg^−1^, available phosphorus 4.7 g kg^−1^, available potassium 10.2 g kg^−1^ and available magnesium 2.2 g kg^−1^ and pH_KCl_ 7.08. The exchangeable cation contents were as follows: sodium 0.3 g kg^−1^, potassium 0.8 g kg^−1^, magnesium 0.5 g kg^−1^ and calcium 2.7 g kg^−1^. Urea was manufactured by EUROCHEM BDG (Poland). Its properties are as follows: molar mass 60.06 g mol^−1^, density 1.32 g cm^−3^ and solubility in water 480 g dm^−3^.

### Experimental Procedure

The experiments were carried out in triplicate under laboratory conditions, with the following variable factors: (a) type of petroleum product. biodiesel, diesel oil, fuel oil and unleaded petrol; (b) degree of contamination of the soil with the petroleum products, 0, 2, 4, 8 and 16 g kg^−1^ DM of soil; (c) type of the biostimulating substance, compost and urea, and (d) incubation time of the soil, 15, 30, 60, 90 and 180 days. One hundred grams of air-dried soil (loamy sand) screened through a sieve with a mesh size of 2 mm was weighed out to each of the beakers with a volume of 150 cm^3^.

In order to neutralise acidification of the soil, it was limed using CaCO_3_ before the experiment, in the amount of 0.18 g kg^−1^ DM of soil. In order to carry out the biostimulation of natural soil microbiota, compost in the amount of 10 g kg^−1^ DM of soil and urea in the amount of 250 mg N kg^−1^ DM of soil were added to the respective soil samples. Soil thoroughly mixed with the petroleum products and, in appropriate objects, with the compost or urea was brought to 50 % of maximum water capacity, protected and put in an incubator with a constant temperature 25 °C and no access to light. During the whole experiment cycle (180 days), the humidity level of the samples was checked once per week, and the water losses were supplemented.

### Determination of the Activity of Soil Dehydrogenases

After 15, 30, 60, 90 and 180 days, the experiments were liquidated and the activity of soil dehydrogenases was determined according to the procedure described in Öhlinger (1996). The determinations were carried out in triplicate. From each soil sample, 6 g of the soil was weighed out 0.06 g CaCO_3_, and 1 cm^3^ 3 % aqueous solution of 1,3,5-phenyl-tetrazolium chloride and 2.5 cm^3^ demineralised water were added to it successively. The samples were incubated for 24 h in 37 °C. After the incubation, 25 cm^3^ methyl alcohol was added to every sample. Then, the content of each sample was thoroughly mixed and transferred quantitatively onto a filtering set consisting of a 50-cm^3^ volumetric flask and a funnel with a quantitative hard filter with a diameter of 9 cm. This operation was repeated twice. After filtering, the flasks were made up to the mark with methyl alcohol, their contents were thoroughly mixed and absorbances were measured on a spectrophotometer Aquarius CE7500 Cecil Instruments, at a wavelength of *λ* = 485 nm. The results were presented as micromoles of triphenylformazan per kilogram DM per hour.

### Determination of the Effect Indices of a Given Petroleum Product and Stimulating Substance

Based on the activity of soil dehydrogenases, indices of the petroleum product effect and stimulating substance effect were calculated using the following formulas:

$$ {\mathrm{IF}}_{\mathrm{pp}}\kern0.5em =\kern0.5em \frac{A_{\mathrm{pp}}}{A_0} $$

IF_pp_: Index of the petroleum product effect
*A*_pp_: Activity of dehydrogenases in the soil contaminated with the petroleum product
*A*_0_: Activity of dehydrogenases in the non-contaminated soil

$$ {\mathrm{IF}}_{\mathrm{b}}=\frac{A_{\mathrm{b}}}{A} $$

IF_b_: Index of the stimulating substance effect
*A*_b_: Activity of dehydrogenases in the soil subjected to biostimulation
*A*: Activity of dehydrogenases in the soil not subjected to biostimulation

If IF = 1, there is no influence of the tested factor on dehydrogenases; IF < 1, there is inhibition of the dehydrogenases activity by the tested factor and IF > 1, there is stimulation of the dehydrogenases activity by the tested factor.

### Statistical Analysis

The results of the studies were developed statistically using a statistical software package STATISTICA v. 12.0 (Statsoft, Inc., Statistica 2014). Based on the analysis of the effect measure *η*^2^ by variance analysis—ANOVA—the percentage shares of all variable factors affecting the activity of dehydrogenases were defined. Homogeneous groups were calculated using Tukey’s test with *P* = 0.01. The diversified influence of the petroleum products on the activity of dehydrogenases was illustrated using the index of the petroleum product effect and that of biostimulation with compost and urea—using the index of the stimulating substance effect.

## Results

### Activity of Dehydrogenases

The activity of dehydrogenases in the soil non-contaminated with petroleum products was in the range of 3.967 μM TFF to 16.414 μM TFF kg^−1^ DM h^−1^, depending on the incubation time (Table 3). In the soil fertilised with compost, it amounted from 9.845 μM TFF to 12.887 μM TFF, while in the soil fertilised with urea—from 0.676 μM TFF to 5.815 μM TFF. On average, irrespective of the test day, the activity in the soil fertilised with compost was 16 % higher, and in the case of urea—72 % lower than that of the non-fertilised soil.

**Table 3:** Activities of dehydrogenases in soil not contaminated with petroleum products (μmol TFF kg^−1^ DM of soil)

Addition	Incubation time, days				
	15	30	60	90	180
0	8.317b	11.720a	16.414a	3.967b	7.651b
Compost	12.887a	10.973b	9.845b	10.575a	11.474a
Urea	5.815c	0.938c	0.676c	2.408c	3.602c

The same letter means a homogeneous group in the columns

Biodiesel (Bd) stimulated the activity of soil dehydrogenases on every test day (Table 4). Over 90 days, the activity decreased, together with an increase in the degree of contamination with Bd. On the 180th day, a decrease in the activity occurred in the soil subjected to the effect of 2 g Bd kg^−1^ DM, while doses of 4–16 g kg^−1^ increased the activity by from 0.660 to 0.799 times. The response of dehydrogenases to contamination with diesel oil (D) was also significant (Table 4). On almost all test days, the activity of these enzymes increased, together with an increase in the contamination degree. Such a tendency lasted for 180 days. However, the stimulating action of the lowest dose (2 g kg^−1^ DM of soil) declined on the 90th and 180th days.

**Table 4:** Index of the influence of petroleum products (IF_pp_) on the activity of soil dehydrogenases

Dose Pp	Incubation time, days				
g kg^−1^ DM of soil	15	30	60	90	180
Biodiesel (Bd)					
2	3.337a	2.008a	1.416a	2.208a	0.886d
4	3.378a	1.982a	1.397a	1.836b	1.660b
8	1.997b	1.531b	1.290b	1.368c	1.763a
16	0.910c	0.933c	0.715c	1.089d	1.799a
Diesel (D)					
2	1.857c	1.533c	1.328b	0.828d	0.966c
4	2.278b	1.559bc	1.313b	1.510c	1.323b
8	2.587a	1.603b	1.484a	1.786b	1.324b
16	2.628a	1.668a	1.298b	2.028a	2.366a
Fuel oil (Fo)					
2	1.806d	1.333c	1.208c	0.742d	1.138c
4	2.221c	1.535b	1.248bc	1.224c	1.218b
8	2.461b	1.572b	1.287b	2.097b	1.459a
16	2.899a	1.819a	1.609a	2.417a	1.494a
Unleaded petrol (P)					
2	0.855a	0.915a	0.997d	0.426a	0.836a
4	0.640b	0.774b	1.102c	0.386b	0.622b
8	0.582c	0.698c	1.286b	0.336c	0.534c
16	0.588c	0.622c	1.374a	0.259d	0.500c

The same letter means a homogeneous group in the columns for a petroleum product (Pp)

Fuel oil (Fo), similarly to biodiesel and diesel oil, stimulated dehydrogenases (Table 4). Its effect decreased more evidently the longer the experiment lasted. On the 180th day, the stimulating effect was exerted only by the dose of 16 g kg^−1^ DM of the soil. Petrol (P) affected dehydrogenases adversely. On almost all test days, the values of the index of the influence of this contaminant on dehydrogenases remained below 1 (Table 4). Values above 1 were observed only on the 60th day for doses of 4–16 g P kg^−1^ DM of the soil.

**Fig. 1:**
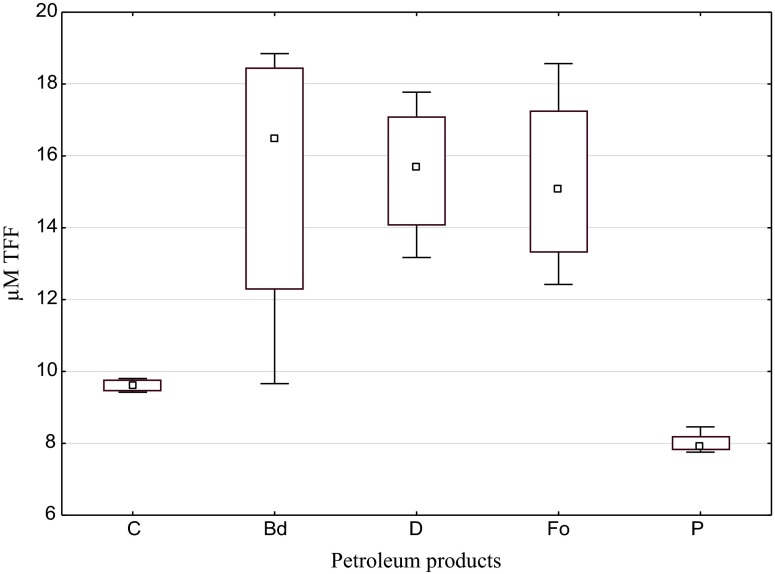
Activity of dehydrogenases in soil contaminated with petroleum products (μM TFF kg^−1^ DM of soil). *C* control, *Bd* biodiesel, *D* diesel oil, *Fo* fuel oil, *P* unleaded petrol

**Fig. 2:**
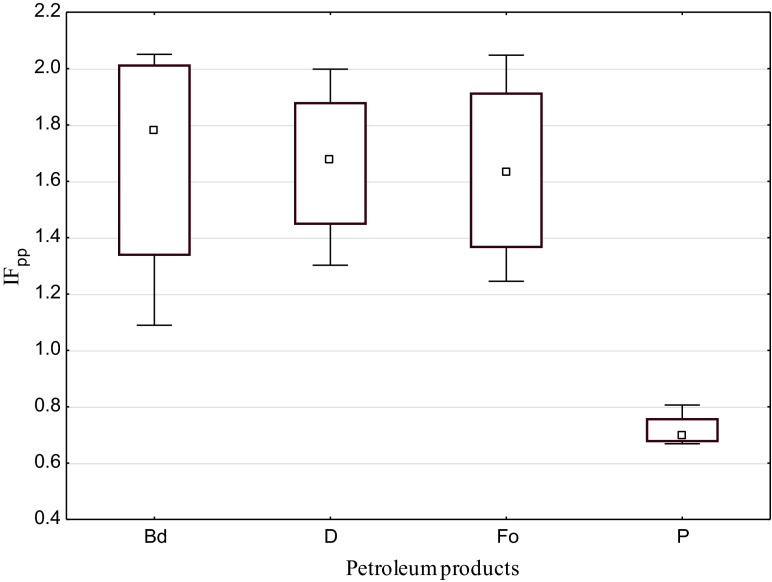
Mean index of the influence of petroleum products (IF_pp_) on the activity of soil dehydrogenases. *Bd* biodiesel, *D* diesel, *Fo* fuel oil, *P* unleaded petrol

Summarising the evaluation of the influence of the tested substances on soil dehydrogenases, one may unequivocally ascertain that biodiesel, diesel oil and fuel oil stimulate these enzymes and petrol inhibits them (Fig. 1). Mean indices of the effect of the first three products were approximate and contained in the range from 1.639 (Fo) to 1.675 (Bd), while in case of petrol, the value of this index was significantly lower than 1 (0.717), proving its inhibitive influence on dehydrogenases (Fig. 2.)

### Biostimulation of the Soil

**Fig. 3:**
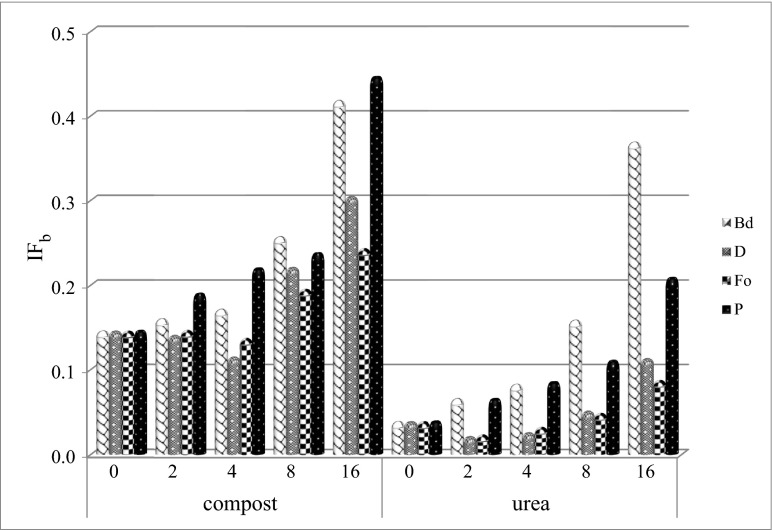
Index of the influence of biostimulation (IF_b_) with compost and urea on the activity of soil dehydrogenases 0, 2, 4, 8 and 16—dose of petroleum products (g kg^−1^ DM of soil). *Bd* biodiesel, *D* diesel, *Fo* fuel oil, *P* unleaded petrol

The biostimulation of the soil with compost affected dehydrogenases favourably (Table 5). In the soil non-contaminated with petroleum products, the compost increased the activity by 45 % on average. The mean index of the effect amounted to 1.45. In the contaminated soils, mean indices calculated from all doses and dates amounted to in the objects from Bd 2.508, D 1.946, Fo 1.796 and P 2.724 (Fig. 3).

**Table 5:** Index of the effect of biostimulation with compost (IF_b_) on the activity of soil dehydrogenases

Dose Pp	Incubation time, days				
g kg^−1^ DM of soil	15	30	60	90	180
Biodiesel (Bd)					
0	1.549c	0.936d	0.600e	2.666cd	1.500d
2	1.124d	0.870d	0.779d	1.909d	3.279b
4	1.308d	1.339c	1.065c	2.857c	1.954c
8	2.610b	2.043b	1.479b	4.762b	1.916c
16	6.026a	3.545a	3.177a	6.058a	2.058a
Diesel (D)					
0	1.549c	0.936c	0.600c	2.666d	1.500c
2	1.395d	0.788d	0.390d	3.081c	1.329d
4	1.301d	1.015c	0.460d	1.925e	1.011e
8	2.041b	2.093b	0.808b	3.837b	2.235a
16	2.945a	3.229a	1.928a	5.035a	2.067b
Fuel oil (Fo)					
0	1.549c	0.936c	0.600c	2.666d	1.500a
2	1.530c	0.946c	0.346d	3.312e	1.140b
4	1.313d	1.014c	0.639c	2.795c	1.043c
8	2.385b	2.081b	0.932b	3.458b	0.861d
16	3.159a	3.161a	1.188a	3.824a	0.787e
Unleaded petrol (P)					
0	1.549d	0.936d	0.600d	2.666e	1.500d
2	2.031c	1.535a	0.639d	3.620d	1.615c
4	2.623a	1.528a	0.687c	4.208c	1.890b
8	2.193b	1.468b	0.862a	5.269b	2.048a
16	2.129bc	1.257c	0.759b	16.058a	2.055a

The same letter means a homogeneous group in the columns for a petroleum product (Pp)

The biostimulation of the soil with urea (Table 6) did not prove as effective as in the case of compost. In the non-contaminated soil, indices lower than 1 on all test days prove the negative influence of this fertiliser on dehydrogenases. In the contaminated soils, the effect of urea depended on the type of petroleum product. In the soil with biodiesel, the mean index of the urea effect amounted to 1.681, diesel oil 0.520, fuel oil 0.469 and unleaded petrol 1.165 (Fig. 3).

**Table 6:** Index of the effect of biostimulation with urea (IF_b_) on the activity of soil dehydrogenases

Dose Pp	Incubation time, days				
g kg^−1^ DM of soil	15	30	60	90	180
Biodiesel (Bd)					
0	0.699e	0.080e	0.041c	0.607d	0.471c
2	0.917d	0.434d	0.012c	0.389e	1.492a
4	1.182c	0.803c	0.222b	1.089c	0.799b
8	2.678b	1.745b	0.279b	2.403b	0.770b
16	5.919a	3.838a	1.945a	5.228a	1.484a
Diesel (D)					
0	0.699cd	0.080b	0.041a	0.607b	0.471b
2	0.576d	0.029c	0.016c	0.125d	0.281d
4	0.743c	0.032c	0.018c	0.083d	0.373c
8	1.558b	0.066bc	0.019c	0.243c	0.621a
16	2.986a	0.617a	0.028b	1.602a	0.388c
Fuel oil (Fo)					
0	0.699d	0.080b	0.041a	0.607a	0.471c
2	0.680d	0.035c	0.015c	0.176c	0.214d
4	0.934c	0.039c	0.018bc	0.126cd	0.444c
8	1.469b	0.055bc	0.022b	0.075d	0.761b
16	2.566a	0.120a	0.019bc	0.288b	1.334a
Unleaded petrol (P)					
0	0.699e	0.080c	0.041b	0.607e	0.471b
2	0.925d	0.818b	0.030b	1.264c	0.182c
4	1.247c	0.896ab	0.054b	1.543bc	0.479b
8	1.397b	0.877ab	0.325a	1.961b	0.917a
16	1.522a	0.912a	0.399a	6.443a	1.102a

The same letter means a homogeneous group in the columns for a petroleum product (Pp)

The data presented in Table 7 indicate unequivocally that the activity of dehydrogenases varied over time. Also, it depended on the biostimulation applied. The effects of biodiesel, diesel oil and fuel oil were mostly determined by the degree of soil contamination. The share of this factor in the formation of dehydrogenase activity ranged from 21.30 % (Fo) to 26.90 % (D). On the other hand, the dose of petrol did not affect dehydrogenases significantly, while compared to other variables, its effect was limited to 1.08 %. The biostimulation of the soil with compost and urea affected dehydrogenases significantly. The share of this factor in the formation of the activity ranged from 17.17 % (D) to 30.69 % (P) depending on the contamination type. Also, the incubation time of the soil affected these enzymes significantly. This independent variable determined the activity of dehydrogenases in the range from 14.39 % (D) to 23.31 % (P).

**Table 7:** Participation of variable factors in the formation of dehydrogenases activity in percentage

Independent variable	Biodiesel (Bd)	Diesel (D)	Fuel oil (Fo)	Unleaded petrol (P)
Biostimulation (a)	17.17	20.55	18.95	30.69
Dose (b): Bd, D, Fo, P	25.30	26.90	21.30	1.08
Time (c)	20.69	14.39	18.13	23.31
Biostimulation · dose (ab)	16.00	11.57	8.30	2.56
Biostimulation · time (ac)	8.00	9.53	11.87	24.92
Dose · time (bc)	4.88	6.89	10.21	11.53
Biostimulation · dose · time (abc)	5.70	6.41	8.11	3.39
Error	0.03	0.03	1.85	1.54

## Discussion

### Activity of Dehydrogenases

The activity of dehydrogenases is one of the most frequently used biological parameters for the evaluation of soil quality. Being intracellular enzymes, dehydrogenases are often mentioned as a good parameter for the estimation of contaminants (Galiulin et al. 2012; Lipińska et al. 2014). They perform as indicators of contamination of the environment with lipophilic substances because their presence reduces the redox potential as a result of the modification of oxygenation conditions, leading to a change in the terminal acceptor from oxygen molecule to Fe^3+^ cations. These are additional factors shaping the diversity of soil microorganisms. According to Galiulin et al. (2012), the degradation rate of hydrocarbons found in oil products increases with an increase in the activity of dehydrogenases which are directly involved in this process. Comparing the effect of diesel oil and biodiesel, the authors of Lapinskiené et al. (2006) demonstrated a statistically significant dependence between the increasing diesel oil dose and the reduction of dehydrogenase activity. They strove to prove unequivocally the toxicity of the tested petroleum products. In our own studies, the influence of petroleum products on the activity of dehydrogenases depended strictly on their type. The activity of the analysed oxidoreductases was positively correlated with the degree of contamination of the soil with biodiesel, diesel oil and fuel oil and negatively with unleaded petrol. The stimulating influence of diesel oil and fuel oil on the activity of dehydrogenases is indicated by the studies of Kucharski and Jastrzębska (2006) and Wyszkowska et al. (2002). The high activity of these enzymes may result from an intensified growth of microorganisms and their increased activity, because diesel oil may be a good nutrient for some microorganisms (Kucharski and Jastrzębska 2005; Wyszkowska and Kucharski 2005). It is noteworthy that a mixture of PAH petroleum products affects the enzymes still less adversely than PAH penetrating the environment in their pure state, e.g. naphthalene, phenanthrene, anthracene and pyrene (Lipińska et al. 2014). The negative influence of polycyclic aromatic hydrocarbons on the activity of dehydrogenases was reported in Gianfreda et al. (2005). They proved this on the basis of studies on samples of soil exposed to contamination by crude oil for 50 years. This report emphasises the particular susceptibility of dehydrogenases to the presence of petroleum products in the soil. Our studies lasted for only 180 days, but even during this period, significant changes in the influence of the individual petroleum products on dehydrogenase activity could be observed. As the deposition of petroleum products was one time at the start of the experiment, the tendency of a reducing pressure of all petroleum products on dehydrogenases could be observed. This could result from a partial biodegradation of hydrocarbons by soil microbes (Xia et al. 2015). The possibility to stimulate indigenous microorganisms by some petroleum-derived contaminations is indicated by Wu et al. (2014).

### Biostimulation of the Soil

Supplementation of the soil with organic matter, as a repository of an available pool of organic compounds, is key during the adaptation of the ecosystem to unfavourable environmental conditions. Stimulation of the biochemical activity by the application of various organic compounds is discussed in numerous scientific papers (Tejada et al. 2008; Wyszkowska and Wyszkowski 2010; Sayara et al. 2010; Galiulin et al. 2014). Their authors try to use various substances as stimulants for the indigenous microorganisms. The proposed solutions include the application of compost (Mehta et al. 2014; Wyszkowska and Wyszkowski 2006; Wyszkowski and Ziółkowska 2013), urea (Komilis et al. 2009; Wyszkowska et al. 2006), finely ground straw, sawdust (Wyszkowska et al. 2002), biological preparations (Galiulin and Galiulina 2015) and whey (Jonsson and Östberg 2011). In a study by Galiulin and Galiulina (2015), biocompost and biopreparations enabled almost complete degradation of hydrocarbons from crude oil and gas condensate. The effectiveness of remediation was evaluated based on, among others, dehydrogenase activity, and the research findings have been applied in practice (Patent of the Russian Federation, RU2387996 (C1)).

The index representing the effect of stimulating substances (IF_b_) on the activity of soil enzymes in soil contaminated with petroleum products was used in the study. The indices of biostimulation define the stability of the soil system and its production capacity. They also serve as indicators of emerging dangers in the soil. The discussed index clearly illustrates the tendencies of changes in the influence of the analysed substances on soil organisms. Thanks to a simple mathematical construction, there is a possibility to analyse the effects of the applied biostimulators and petroleum products. In our research, it was proved that compost was useful in the biostimulation of soils contaminated with petroleum products. It increased the activity of dehydrogenases both in contaminated and non-contaminated soils. Therefore, one may conclude that compost can accelerate the microbiological degradation of petroleum-derived contaminants, because it constitutes a source of necessary cosubstrates, nutrients or microorganisms, including the following: mesophilic and thermophilic bacteria, actinobacteria and fungi (Mehta et al. 2014). Galiulin and Galiulina (2015), who investigated the effects of biopreparations in soil contaminated with oil at 50 g kg^−1^, reported a significant increase in dehydrogenase activity. In soil supplemented with Bioros (0.5 g kg^−1^), which contains two groups of microorganisms: *Rodococcus* sp. and *Candida* sp. at 10^10^ cell g^−1^, dehydrogenase activity was 19-fold higher than in oil-contaminated soil that was not supplemented with the above biopreparation. In soil treated with the Piksa biopreparation (100 g kg^−1^), composed of a turf-manure mixture and enriched with hydrocarbonoxidising microorganisms in the quantity of 10^6^ cells g^−1^, dehydrogenase activity increased 11-fold. The noted increase in dehydrogenase activity after the application of biopreparations is indicative of microbial degradation of hydrocarbons from crude oil. This is confirmed by the studies of Xia et al. (2015), indicating that bioaugmentation connected with biostimulation yields a better effect in the degradation of hydrocarbons of petroleum products than bioaugmentation alone. Moreover, thanks to its structure, compost may improve the physical properties of the soil, changing its pH, water capacity and structure (Semple et al. 2001). The favourable influence of compost on dehydrogenases activity observed in our studies is also confirmed by the investigations of Sayara et al. (2010), where the biochemical activity of soils contaminated with PAH was analysed.

In our studies, urea was decidedly less useful than compost in the biostimulation of soils contaminated with petroleum products. The index of the effect of biostimulation with urea (IF_b_) on the activity of soil dehydrogenases in the non-contaminated soil and in the soil contaminated with fuel oil and diesel oil assumed values lower than 1, while in soil contaminated with biodiesel and petrol—values higher than 1. These low values of the IF_b_ indices may prove the lack of dependence between the attempt to compensate the C:N ratio and acceleration of the process of petroleum hydrocarbon degradation in the studied soil. Such a state may be a result of a change in the air-water conditions of the soil environment.

The small effect of nitrogen supplementation of the soil contaminated with petroleum-derived hydrocarbons is also proved by the studies Bento et al. (2005), Aspray et al. (2008) and Komilis et al. (2009). According to Lamy et al. (2013), the supplementation is favourable only when the cell can use its enzymes of the metabolic pathways of petroleum hydrocarbon degradation for decomposition of the stimulating substance. Also, the form of the nitrogen applied is of great importance. Inorganic nitrogen is better. According to Komilis et al. (2009), the application of nitrogen in the form of NH_4_Cl is more favourable than the use of urea.

## Conclusions

The petroleum products affect the soil dehydrogenases in various ways. Biodiesel, diesel oil and fuel oil stimulate these enzymes, while petrol acts as an inhibitor. Among the substances tested regarding biostimulation of soils contaminated with petroleum products, compost is definitely more useful than urea, and therefore, the former should be used for the remediation of such soils. Stimulation of dehydrogenases by compost, both in contaminated and non-contaminated soils, proves that it can accelerate the microbiological degradation of petroleum-derived contaminants.
